# Dual Function of the NK Cell Receptor 2B4 (CD244) in the Regulation of HCV-Specific CD8+ T Cells

**DOI:** 10.1371/journal.ppat.1002045

**Published:** 2011-05-19

**Authors:** Verena Schlaphoff, Sebastian Lunemann, Pothakamuri Venkata Suneetha, Jerzy Jaroszewicz, Jan Grabowski, Julia Dietz, Fabian Helfritz, Hueseyin Bektas, Christoph Sarrazin, Michael Peter Manns, Markus Cornberg, Heiner Wedemeyer

**Affiliations:** 1 Department for Gastroenterology, Hepatology and Endocrinology, Hannover Medical School, Hannover, Germany; 2 J. W. Goethe University Hospital, Frankfurt am Main, Germany; 3 Department for Visceral Surgery, Hannover Medical School, Hannover, Germany; Nationwide Children's Hospital, United States of America

## Abstract

The outcome of viral infections is dependent on the function of CD8+ T cells which are tightly regulated by costimulatory molecules. The NK cell receptor 2B4 (CD244) is a transmembrane protein belonging to the Ig superfamily which can also be expressed by CD8+ T cells. The aim of this study was to analyze the role of 2B4 as an additional costimulatory receptor regulating CD8+ T cell function and in particular to investigate its implication for exhaustion of hepatitis C virus (HCV)-specific CD8+ T cells during persistent infection. We demonstrate that (i) 2B4 is expressed on virus-specific CD8+ T cells during acute and chronic hepatitis C, (ii) that 2B4 cross-linking can lead to both inhibition and activation of HCV-specific CD8+ T cell function, depending on expression levels of 2B4 and the intracellular adaptor molecule SAP and (iii) that 2B4 stimulation may counteract enhanced proliferation of HCV-specific CD8+ T cells induced by PD1 blockade. We suggest that 2B4 is another important molecule within the network of costimulatory/inhibitory receptors regulating CD8+ T cell function in acute and chronic hepatitis C and that 2B4 expression levels could also be a marker of CD8+ T cell dysfunction. Understanding in more detail how 2B4 exerts its differential effects could have implications for the development of novel immunotherapies of HCV infection aiming to achieve immune control.

## Author Summary

Infection with the hepatitis C Virus (HCV) is a world-wide health burden, the infection becomes persistent in the majority of cases. In chronic patients HCV-specific immune responses are weak, HCV-specific CD8+ T cells were shown to be functionally exhausted and to be negatively controlled by costimulatory molecules like PD-1. Here, we show that the costimulatory molecule 2B4 (CD244) is also involved in the regulation of HCV-specific CD8+ T cell responses and that 2B4 expression is selectively upregulated on virus-specific CD8+ T cells in persistent infections. Proliferation of HCV-specific CD8+ T cells from chronic patients increased by 2B4 cross-linking only if the *ex vivo* 2B4 expression was low, while we could observe no effect on samples with high 2B4 expression levels. Of note, expression of the intracellular 2B4 adaptor molecule SAP, which leads to an activation of the cell, decreased with higher 2B4 expression levels. Finally, we were able to show that 2B4 cross-linking can counter-act enhanced proliferation of HCV-specific CD8+ T cells seen upon PD-1 blockade. Thus, our study provides new insights into the regulation of CD8+ T cell responses demonstrating an implication of the costimulatory molecule 2B4.

## Introduction

The outcome of viral infections is dependent on the function of CD8+ T cells. The activity of CD8+ T cells is tightly regulated by costimulatory molecules which are expressed on the cell surface. Upon interaction with their respective counterparts, various intracellular signalling pathways can be modified leading to altered effector functions [1].

Infection with the hepatitis C virus (HCV) results in persistent infection in the majority of cases [2]. The mechanisms leading to chronicity are yet poorly understood. Besides viral escape mutations HCV-specific CD8+ T cells are functionally impaired and lack key effector functions such as cytokine production, proliferation and cytotoxicity [3], [4]. Virus-specific CD8+ T cell responses generated during the early onset of HCV infection are strong and multispecific, however, in settings of persistent virus infections virus-specific T cells gradually become exhausted [5], [6]. The mechanisms leading to exhaustion of T cells are only partially understood, beside changes in the cytokine milieu and the lack of CD4+ T cell help [7], [8], altered expression levels of coinhibitory molecules may also be of importance. In mouse models of persistent viral infections exhaustion of virus-specific CD8+ T cells was shown to be linked to the expression of the coinhibitory molecule PD1 [9], [10]. Subsequently, also in human chronic viral infections impaired CD8+ T cell functions have been reported to be associated with PD1 expression [5], [11], [12]. However, the susceptibility to blockade of PD1 signaling varied between individuals and PD1 blockade alone was not able to restore function of intrahepatic HCV-specific CD8+ T cells [13]. Similarly, it was shown that HCV-specific CD8+ T cells in acute hepatitis C can be functional despite continued PD1 expression [14]. These findings implicate that multiple factors might be involved in the control of CD8+ T cell function and establishment of T cell exhaustion. Consequently, studies performed in mouse models with persistent viral infections demonstrated that functionally exhausted cells showed expression of multiple costimulatory molecules [15].

Besides PD1 one of the costimulatory molecules identified being upregulated in exhausted virus-specific CD8+ T cells is the NK cell receptor 2B4 (CD244). This molecule expressed on the cell surface belongs to the family of SLAM-related receptors and contains two cytoplasmatic ITSM (Immunoreceptor Tyrosine-based Switch Motif) [16], which get phosphorylated upon ligation with the high-affinity counterpart CD48 [17]. Initially, 2B4 was described as a costimulatory receptor enhancing NK and CD8+ T cell functions [18], [19], but a recent study showed that 2B4 can elicit both activating as well as inhibitory signals on NK cells [20]. Importantly, the consequence of 2B4 ligation is dependent on the cell surface expression intensity of 2B4 and the relative availability of intracellular adaptor molecules. Interestingly, 2B4 signalling can occur via different adaptor molecules, while the recruitment of SAP (SLAM-associated protein) is supposed to cause an activation of the cell, involvement of the adaptor molecule EAT-2 (EWS-Fli1-activated transcript 2) seems to result in inhibitory signaling [21]. 2B4 has recently been described to be expressed also on HBV- and HCV-specific [22] CD8+ T cells, however, functional consequences of 2B4 stimulation have not been investigated yet.

Due to the costimulatory potential and dual function of 2B4, we aimed to investigate the role of 2B4 for the control of HCV-specific CD8+ T cell function.

## Results

### 2B4 expression on virus-specific CD8+ T cells

**Figure 1:**
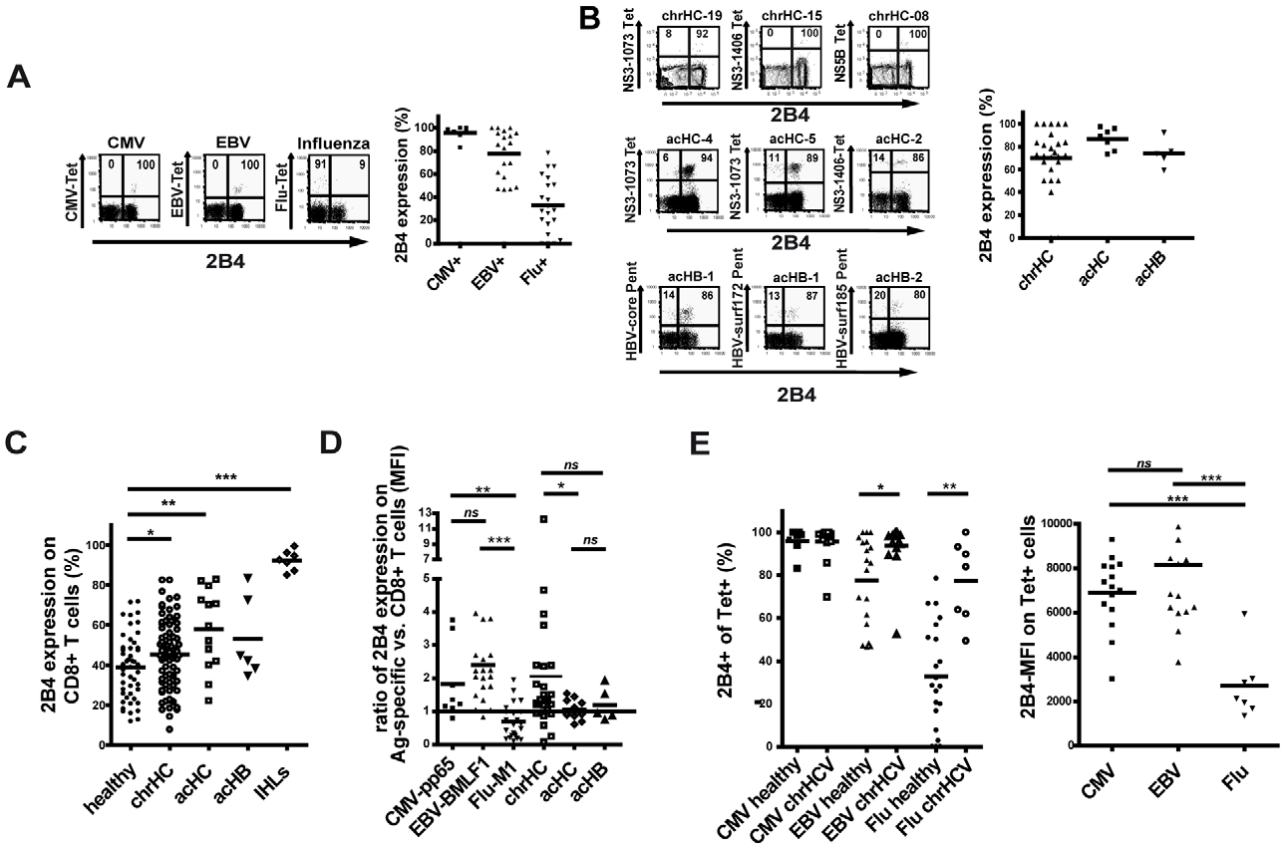
2B4 expression on virus-specific and bulk CD8+ T cells. (**A**) CMV-pp65-, EBV-BMLF1- and Influenza A virus-specific cells from healthy individuals were stained for 2B4 expression. Representative FACS plots are shown on the left, summarized data of frequency of 2B4 expression on virus-specific CD8+ T cells from all individuals analyzed are shown in the plot on the right side. (**B**) Virus-specific CD8+ T cells from patients with chronic hepatitis C (upper left panel; n = 24), acute hepatitis C (middle left panel; n = 7) and acute hepatitis B (lower left panel; n = 5) were stained *ex vivo* for 2B4. High 2B4 expression frequency could be found in all three cohorts. Representative FACS plots of three individuals each are shown on the left side, summarized data of frequency of 2B4 expression on virus-specific CD8+ T cells from all individuals analyzed are shown in the right plot. (**C**) Expression of 2B4 on bulk CD8+ T cells was analyzed *ex vivo* by flow cytometry, values given represent frequency of expression of 2B4 on total CD8+ T cells in percent. PBMC from healthy individuals (closed circles, mean 39% ±16%; n = 48), patients with chronic hepatitis C (open circles, mean 45% ±17%; n = 78) or acute hepatitis C (open squares, mean 58% ±19.5%; n = 13) or acute hepatitis B (open triangles, mean 53% ±18.4%; n = 6) and T cells from healthy liver tissues (open diamonds, mean 87% ±13%; n = 7) were isolated and directly stained for 2B4. Cells were gated on CD14-, CD19- and CD56-negative lymphocytes and CD8+ T cells. 2B4 expression frequency on bulk CD8+ T cells from patients with chronic hepatitis C and acute hepatitis C was found to be significantly increased as compared to healthy controls (p = 0.04 and p = 0.004, respectively). (**D**) Ratio of 2B4 expression levels. Mean 2B4 expression levels on virus-specific T cells was divided by the mean 2B4 expression level on the respective individual's bulk CD8+ T cells in order to calculate the ratio for showing selective change of 2B4 expression. A ratio >1 indicates higher expression on tetramer+ CD8+ T cells and a ratio <1 indicates lower 2B4 expression levels on tetramer+ CD8+ T cells as compared to bulk CD8+ T cells. Ratios were calculated for cells specific for CMV-pp65 (closed squares; mean1.82±1.1; n = 8), EBV-BMLF-1 (closed triangles up; mean 2.4±1.4; n = 19) and Flu-M1 (closed triangles down; mean 0.7±0.5; n = 24) in healthy individuals, for HCV-specific CD8+ in patients with chronic hepatitis C (open squares; mean 2.1±2.4; n = 26) and acute hepatitis C (open diamonds; mean 1.1±0.3; n = 10) and for HBV-specific CD8+ T cells in patients with acute hepatitis B (open triangles up; mean 1.2±0.5; n = 5). Cells were gated on CD14-, CD19- and CD56-negative lymphocytes and CD8+ T cells. (**E**) Expression of 2B4 on CMV-, EBV- and Flu-specific CD8+ T cells in healthy individuals as compared to chronic HCV patients. Analyzing the frequency of 2B4 expression in the two cohorts revealed an elevated frequency of 2B4 expression on Flu-specific T cells in chronic HCV patients as compared to healthy individuals (left plot). However, 2B4 expression levels on Flu-specific CD8+ T cells were lower than on CMV- and EBV-specific T cells as seen before in healthy individuals.

As 2B4 can be expressed not only by NK cells but also by CD8+ T cells we first aimed to investigate if 2B4 is also detectable on virus-specific CD8+ T cells in latent infections (CMV, EBV) and after resolved infections (Influenza A). As shown in Figure 1a, we observed a distinct pattern of 2B4 expression on these virus-specific CD8+ T cells in healthy individuals. While CMV- and EBV-specific CD8+ T cells displayed a high frequency of 2B4 expression (CMV: mean 96% ±5.6%; EBV: mean 79% ±18.7%, respectively), only a proportion of Flu-specific CD8+ T cells were positive (mean 29% ±21.9%; see Figure 1a). 2B4 expression levels were also lower on Flu-specific than on CMV- or EBV-specific CD8+ T cells (average 2B4 MFI 25 ±16.6 vs. 163 ±14.9 and 88 ±38.0, respectively; data not shown).

### Expression of 2B4 on CD8+ T cells in viral hepatitis

We next asked for the expression of 2B4 on virus-specific CD8+ T cells in both patients with acute viral hepatitis and in patients with chronic hepatitis C. HCV and HBV-specific CD8+ T cells during acute symptomatic infection showed a high frequency of 2B4 expression (mean 85% ±10.3% and 74% ±10.7; average MFI 97 ±38.9 and 83 ±36.9, respectively; see Figure 1b). On HCV-specific CD8+ T cells from patients with persistent HCV infection the frequency of 2B4 expression was slightly lower than in acute patients, here about three quarter of virus-specific CD8+ T cells expressed 2B4 with a considerable inter-individual variability (mean 70% ± 26%, average MFI 108 ±60.1). The level of 2B4 expression on HCV-specific CD8+ T cells in patients with chronic hepatitis C seemed to be lower in subjects showing viral sequence variants in the respective epitopes. However, in those individuals where the viral sequence was matching to the peptide sequences used not only high 2B4 MFIs could be observed on virus-specific CD8+ T cells, but also low expression levels of 2B4 could be found (see online Supplementary Table S1).

In order to assess the frequency of 2B4 expression on bulk CD8+ T cells we screened PBMCs e*x vivo* for 2B4. We therefore used PBMCs obtained from patients with chronic hepatitis C infection, patients with acute symptomatic hepatitis C and hepatitis B virus infection and analyzed 2B4 expression by flow cytometry in comparison to samples from healthy individuals. Generally, the frequency of 2B4 expression on bulk CD8+ T cells showed a large inter-individual variability in all cohorts analyzed (Figure 1c). In all patient cohorts a higher frequency of 2B4 expression on CD8+ T cells as compared to healthy controls could be found (acHC: mean 58% ±19.5%; acHB: mean 53% ±18.4%; chrHC: mean 45% ±17.1% and healthy: mean 39% ±16%; respectively; see Figure 1c). No correlations of 2B4 expression frequency on total CD8+ T cells with viral load, AST or ALT levels or other clinical marker of liver disease could be seen (data not shown). In addition, 2B4 expression was studied on liver-infiltrating CD8+ T cells from healthy liver tissues which stained highly 2B4-positive in 85% to 98% of cases (mean 79% ±17.5%, see Figure 1c) with higher relative levels of 2B4 expression (average MFI 214±66.7, data not shown) as compared to peripheral lymphocytes (Figure 1c).

### Upregulation of 2B4 expression levels in persistent infections

To investigate upregulation of 2B4 expression on tetramer+ T cells as compared to the respective bulk CD8+ T cells, we calculated a ratio of 2B4 expression levels by dividing the 2B4 MFI on tetramer+ by the 2B4 MFI on bulk CD8+ cells from the respective individual. A ratio >1 indicates higher expression and a ratio <1 indicates lower 2B4 expression on tetramer+ CD8+ T cells as compared to bulk CD8+ T cells of each respective individual. Of note, in healthy individuals the level of 2B4 expression on CMV- and EBV-specific CD8+ T cells showed a selective upregulation as compared bulk CD8+ T cells (mean ratio MFI CMV+: 1.83±1.1 and mean ratio MFI EBV+: 2.4±1.4, respectively, see Figure 1d). This was not the case, however, for Flu-specific CD8+ T cells which in the majority of cases showed lower levels of 2B4 expression as compared to the respective bulk CD8+ T cells (mean ratio MFI Flu+: 0.7±0.5). Importantly, the level of 2B4 expression was selectively increased on HCV-specific CD8+ T cells as compared to the respective bulk CD8+ T cells (mean ratio MFI chrHC 2.1±2.4; see Figure 1d) in chronic hepatitis C. In contrast, virus-specific CD8+ T cells from patients with acute HCV or HBV infection showed almost equal 2B4 expression intensities as compared to the respective bulk CD8+ T cells (mean ratio MFI acHC 1.1±0.3 and mean ratio MFI acHB 1.2±0.5, respectively). Interestingly, the rank order for 2B4 expression between different groups was different between MFI ratios and frequency of 2B4+ cells. For further analysis, grouping in 2B4 high versus 2B4 low expression was performed based on the expression level of 2B4 on tetramer-positive cells (2B4 MFI).

**Figure 2:**
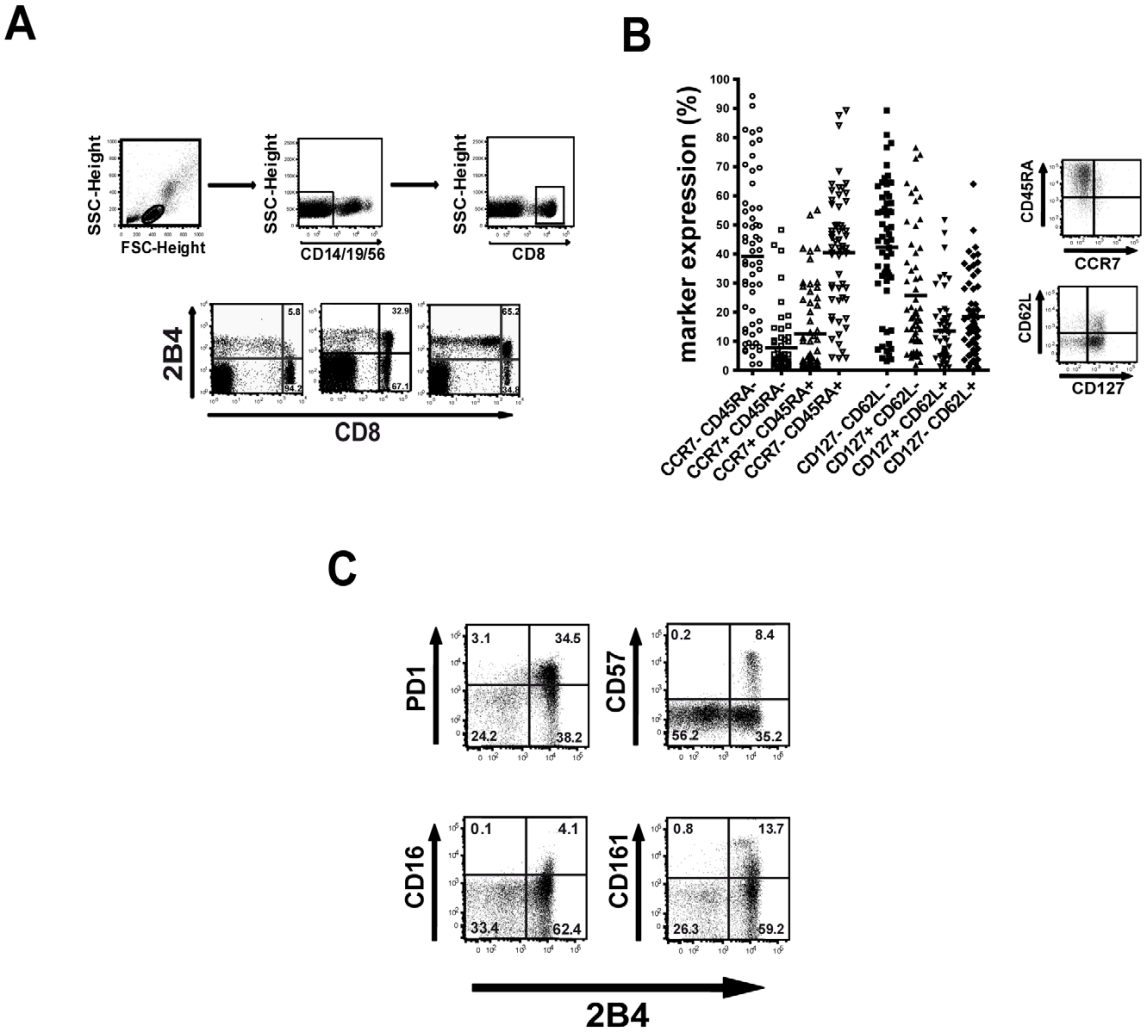
Characteristics of 2B4+ CD8+ T cells. (**A**) For analysis of the phenotype of 2B4+ CD8+ T cells were gated as demonstrated on live cells and after exclusion of CD14/CD19/CD56+ cells (upper panel). Exemplary staining of 2B4 expression on CD8+ T cells in healthy individuals is shown in the lower panel. (**B**) The maturation status of 2B4+CD8+ T cells was analyzed by costaining with CCR7, CD45RA, CD62L and CD127. The frequencies of expression of these markers are shown in the left Figure. Expression showed a high inter-individual variability (CCR7+ CD45RA-: mean 40.5% ±20.9%;CCR7+ CD45RA+: mean 12.6%±14.8%; CCR7- CD45RA+: mean 7.7% ±10.6%; CCR7- Cd45RA-: mean 39.2±25.7; CD62L+ CD127-: mean 25.7% ±20.8%; CD62L+ CD127+: mean 13.4% ±11.4%; CD62L- CD127+: mean 18.5% ±13.9%; CD62L- CD127-: mean 42.3% ±22.3%) and expression of 2B4 on CD8+ T cells could not be linked to a specific memory population subtype. Representative FACS plots are shown on the right side. Cells were gated on CD14/CD19/CD56-negative cells and 2B4+CD8+ T cells. (**C**) Coexpression of 2B4 with the coinhibitory molecule PD1, CD57, CD16 and CD161. Cells were stained for 2B4 together with PD1, CD57, CD16 or CD161, plots are gated on CD14/CD19/CD56-negative lymphocytes. All CD8+ T positive for either PD1, CD57, CD16 or CD161 were found to simultaneously be also positive for 2B4 as shown in the representative FACS plots.

We next investigated 2B4 expression on CMV-, EBV- and Flu-specific CD8+ T cells in patients with chronic hepatitis C. A higher frequency of 2B4 expression on Flu-specific cells was detected as compared to healthy individuals (Figure 1e, left panel) while no difference was seen for CMV- or EBV-specific CD8+ T cells. The level of 2B4 expression (2B4 MFI), however, was lower on Flu-specific T cells as compared to CMV- and EBV-specific cells (Figure 2e, right panel), thus showing the same pattern as seen in healthy individuals.

### Phenotype of 2B4+ CD8+ T cells

To evaluate whether 2B4 expression is linked to a certain phenotype we analyzed the memory status of 2B4+ CD8+ T cells (Figure 2a) by costaining for common differentiation and costimulatory markers. As demonstrated in Figure 2b, 2B4+ CD8+ T cells show a trend towards an elevated coexpression of CD45RA (mean 57% ±23%) and reduced coexpression of CCR7 (mean 25.5% ±26%). Thus, 2B4+ CD8+ T cells preferentially show an effector or effector memory phenotype. Similar patterns were seen using CD127 and CD62L (CD62L+ CD127-: mean 25.7% ±20.8%; CD62L+ CD127+: mean 13.4% ±11.4%; CD62L- CD127+: mean 18.5% ±13.9%; CD62L- CD127-: mean 42.3% ±22.3%). However, as expression levels of these markers show a high inter-individual variability there was no clear link to any memory population subtype.

Costaining of 2B4+ CD8+ T cells with PD1 and CD57 showed a clear correlation of expression of these costimulatory molecules with all PD1+ CD8+ T cells being also positive for 2B4 (Figure 2c). Of note, this was not the case *vice versa* as not all 2B4+ CD8+ T cells also showed expression of PD1. Similar patterns of 2B4-coexpression could be observed with other costimulatory molecules such as CD16 or CD161, where expression of these markers was also always associated with 2B4 expression.

### Consequences of 2B4 cross-linking for CD8+ T cell effector functions

To investigate whether 2B4 has potential costimulatory effects on bulk and virus-specific CD8+ T cells different effector functions of CD8+ T cells were analyzed after 2B4 stimulation. Therefore, the anti-2B4 antibody clone C1.7 was used which is known to activate 2B4 by cross-linking [18]. Of note, anti-2B4 alone without further stimulation did not cause any alteration of T cell proliferation (data not shown) supporting the concept that 2B4 acts as a costimulatory molecule modifying T cell responses to antigenic stimulation. We generated *in vitro* cultures using PBMCs and stimulated with anti-CD3/CD28 or virus-derived peptides and with or without addition of anti-2B4 antibody in the cell culture.

**Figure 3:**
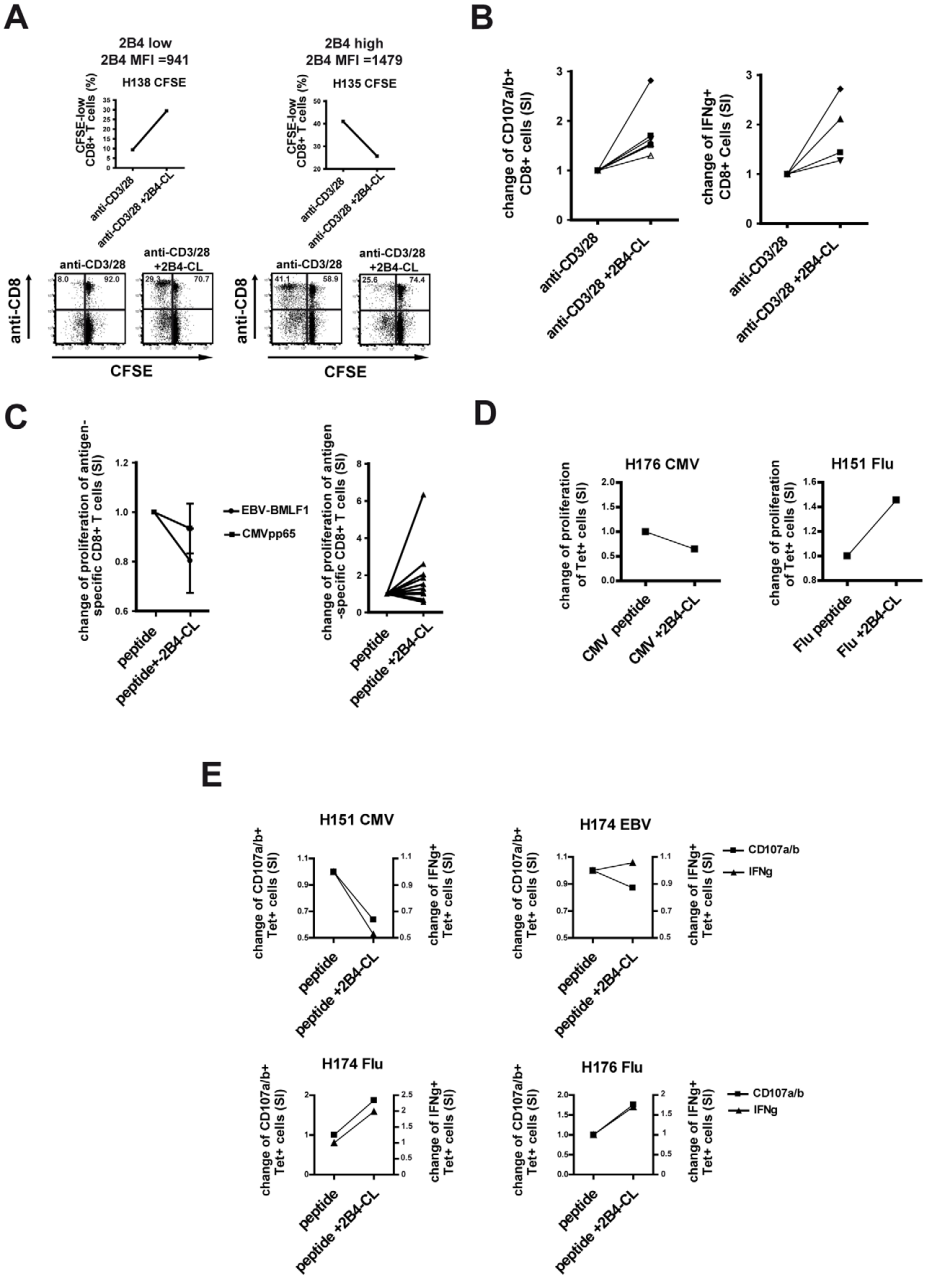
Alteration of CD8+ T cell effector function upon 2B4 cross-linking. (**A**) Bulk CD8+ T cells from healthy individuals showed an increase in proliferation upon anti-CD3/28 stimulation and additional 2B4 cross-linking as compared to anti-CD3/28 stimulation alone. This, however, was only the case for cells with a low 2B4 expression level *ex vivo* (left plots), while cells with high 2B4 expression level *ex vivo* could not be further enhanced by 2B4 cross-linking (right plots). *Ex vivo* 2B4 expression levels are indicated above the respective plot. Frequencies of CFSE-low CD8+ T cells are given, representative plots are shown. (**B**) Additionally, the anti-CD3/28-induced degranulation (as analyzed by CD107a/b expression, left plot) and the IFNγ production (right plot) of bulk CD8+ T cells from healthy individuals with low *ex vivo* 2B4 expression levels could be enhanced by 2B4 cross-linking in several individuals. Stimulation indices (SI) are shown referring to anti-CD3/28 stimulation alone. (**C**) Expansion of antigen-specific CD8+ T cells upon peptide-specific stimulation could not be enhanced by additional 2B4-cross-linking in the case of (2B4 high expressing) CMV- or EBV-specific CD8+ T cells (left plot). However, expansion of (low 2B4 expressing) Flu-specific T cells increased upon additional 2B4 cross-linking in most individuals analyzed (right plot). Stimulation indices are shown referring to peptide stimulation alone. (**D**) Expansion of Flu-specific CD8+ T cells induced by peptide stimulation and additional 2B4 cross-linking is due to an increased proliferation of antigen-specific T cells as seen by a higher frequency of CFSE-low cells. In contrast, CMV- or EBV-specific T cells did not respond to additional 2B4 cross-linking. (**E**) Degranulation (squares) and IFNγ production (triangles) of CMV- or EBV-specific T cells from healthy individuals did not increase upon 2B4 cross-linking in addition to peptide stimulation (upper panels). In contrast, these effector functions increased upon peptide stimulation and simultaneous 2B4 cross-linking in the case of Flu-specific T cells (lower panels).

In line with the report by Chlewicki et al. [20], where the outcome of 2B4 ligation was described to be dependent on the surface expression intensity of 2B4, we observed a difference in the proliferation of cells with high or low 2B4 expression levels directly *ex vivo*. While 2B4 cross-linking enhanced proliferation of CD3/CD28-stimulated 2B4-low cells in a 7 day CFSE assay, no such effect could be observed for cells with high 2B4 expression (Figure 3a). Similarly, other effector functions like degranulation as a marker for cytotoxicity and IFNγ production of bulk CD8+ T cells increased after anti-CD3/CD28 stimulation and additional 2B4 cross-linking only in 2B4-low expressing cells (Figure 3b). Again, this was not the case for samples with high 2B4 expression. However, not all samples with low 2B4 expression levels increased in their effector functions after 2B4 cross-linking.

The functional importance of 2B4 expression for CD8+ T cells further became evident when stimulating sorted 2B4+ and 2B4- CD8+ T cells. In this case, only the 2B4+ T cells responded to TCR-stimulation with an increased degranulation (see online supplementary Figure S1).

The findings on altered effector functions of anti-CD3/28-stimulated bulk CD8+ T cells were confirmed for antigen-specific CD8+ T cells. High 2B4-expressing CMV- and EBV-specific CD8+ T cells showed no increase and in some cases even a decrease of expansion of tetramer positive cells after peptide-specific stimulation and additional 2B4 cross-linking (CMV mean SI = 0.93 +/− 0.2 and EBV mean SI = 0.8 +/− 0.3, respectively; see Figure 3c, left side). In contrast, 2B4-low Flu-specific CD8+ T cells showed an elevated peptide-induced proliferation upon simultaneous 2B4 cross-linking as compared to peptide stimulation alone (mean SI =  1.66 +/− 1.48, Figure 3c, right side). These observations could be confirmed by using the CFSE assay as readout showing an increased proliferation of Flu-specific but not CMV- or EBV-specific CD8+ T cells. These experiments confirmed an increased proliferation of antigen-specific T cells and not only a relative enrichment in cultures (Figure 3d). Similarly, degranulation and IFNγ production of Flu-specific T cells could be enhanced through 2B4 cross-linking, while CMV- or EBV-specific T cells did not respond or showed decreased of effector functions upon 2B4 stimulation (Figure 3e).

Of note, cross-linking of 2B4 using a monoclonal antibody had no impact on the survival and viability of cells. No increase in Annexin-V positive cells was observed when treating cells with anti-2B4 and with or without additional anti-CD3/28 stimulation of the cells (see online supplementary Figure S2). Also, after *in vitro* culture no differences in cell viability between the different cell culture conditions could be seen as analyzed by flow cytometry (according to “live gate”, data not shown).

### Importance of 2B4 for HCV-specific CD8+ T cell expansion

Persistent HCV infection is characterized by the functional exhaustion of the HCV-specific CD8+ T cells. In settings of persistent infection PD1 was shown to be upregulated contributing to the dysfunctionality of these cells. As we could show that 2B4 expression is selectively upregulated on HCV-specific CD8+ T cells, we next wanted to investigate the impact of 2B4 on the function of HCV-specific CD8+ T cells in persistent HCV infection.

**Figure 4:**
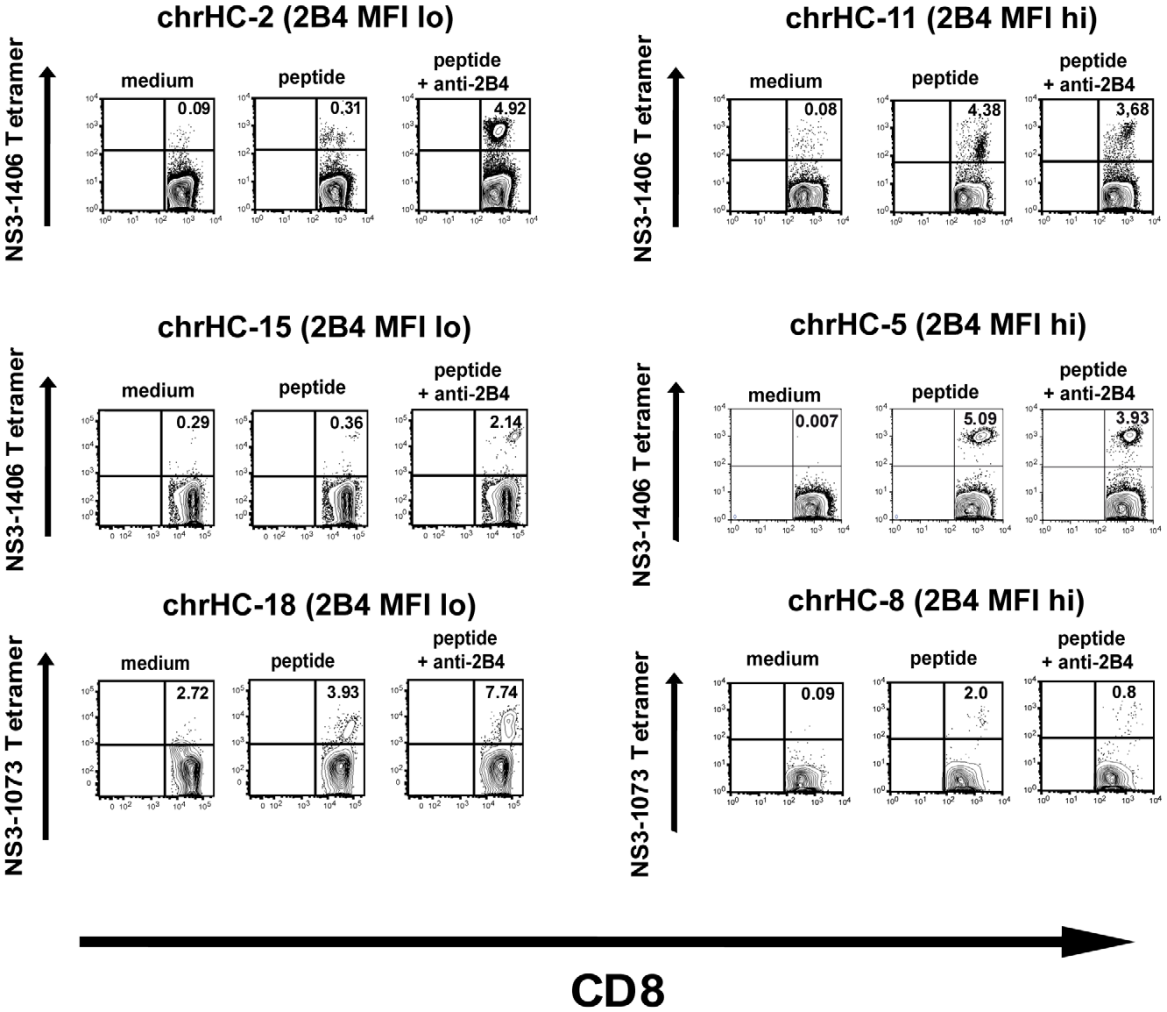
Effect of peptide stimulation and simultaneous 2B4 cross-linking on the expansion of HCV-specific CD8+ T cells in patients with chronic hepatitis C. Exemplary FACS plots from six different day10 cell lines analyzed are shown, frequency of HCV-tetramer+ cells of total CD8+ T cells are indicated. Samples were divided according to the direct *ex vivo* expression intensity (MFI) of 2B4 on HCV-specific CD8+ T cells into 2B4 MFI low (left panel) and 2B4 MFI high (right panel). Cross-linking of 2B4 resulted in an increased frequency of HCV-specific CD8+ T cells in those samples with low 2B4 expression *ex vivo*, while for those samples with high expression no increase or even a decrease of proliferation was observed.

We therefore studied peptide-specific proliferation of HCV-specific CD8+ T cells in a 10 days *in vitro* culture experiment and determined the expansion of tetramer-positive cells. Overall, 86 patients with chronic hepatitis C were screened for HLA-A2 and 39 cell lines were established. 24 cell lines showed detectable tetramer-positive cells and enriched HCV-specific CD8+ T cells could be detected in 19 (79%) cell lines in at least one of the culture conditions. 10 cell lines responded to peptide stimulation alone (see Table 1). Overall, the effects of cross-linking 2B4 varied between individuals. Additional stimulation of 2B4 resulted in an enrichment of HCV-specific CD8+ T cells in 5 individuals (26%) (stimulation index referring to peptide stimulation alone). Interestingly, all of these five samples responding to 2B4 stimulation displayed low 2B4 expression levels on the respective virus-specific cells *ex vivo* (see table 1, p = 0.052 comparing 2B4-low versus 2B4-high tetramer-positive samples). Examples of different cell lines are shown in Figure 4. In addition there was a trend of reduced responsiveness to peptide stimulation alone for 2B4-high samples (2/10 versus 8/15 responding cell lines, p = 0.13). A detailed listing of all cell lines analyzed including the respective *ex vivo* frequencies of HCV-specific T cells is given in table 1.

**Table 1:** Expansion of HCV-specific CD8+ T cells in chronic hepatitis C patients.

chrHCV patient #	epitope	*ex vivo frequency*	medium	+peptide	+2B4-CL	+PDL-1	+2B4-CL +PDL-1	MFI 2B4 on Tet+
#01	NS3-1073	*0.08*	0.02	0.02	0.01	0.01	0.00	**lo**
#01	NS3-1406	*0.06*	0.03	0.01	0.01	0.01	0.02	**lo**
#02	NS3-1073	*0.026*	0.09	0.31	**4.92**	0.10	0.10	**lo**
#03	NS3-1073	*0.032*	1.64	1.73	1.67	1.75	**23.30**	**hi**
#04	NS3-1073	*0.02*	0.21	**0.33**	0.20	0.13	0.17	**hi**
#05	NS3-1406	*0.09*	0.01	5.09	3.93	**19.90**	1.79	**lo**
#06	NS3-1073	*0.22*	0.16	0.76	1.67	4.71	1.99	**lo**
#07	NS3-1073	*0.02*	0.08	0.02	0.06	**0.08**	0.07	**hi**
#08	NS3-1073	*0.1*	0.09	2.01	0.81	**4.40**	1.92	**lo**
#08	NS5B	*0.13*	0.16	0.28	0.23	**0.34**	0.34	**lo**
#09	NS3-1073	*0.02*	0.85	20.30	6.03	15.70	1.36	**lo**
#10	NS5B	*0.01*	0.03	0.05	0.07	0.03	**1.10**	**lo**
#11	NS3-1406	*0.03*	0.08	4.38	3.68	**5.04**	2.64	**lo**
#11	NS3-1073	*0.09*	0.42	0.37	0.53	**0.87**	0.27	**hi**
#12	core132	*0.01*	0.06	0.04	**0.11**	0.08	0.06	**lo**
#12	NS3-1073	*0.03*	1.23	**22.70**	9.59	15.80	7.35	**hi**
#13	NS3-1073	*0.009*	0.02	0.06	0.08	0.04	0.03	**hi**
#14	NS3-1073	*0.04*	0.06	0.04	0.00	**0.34**	0.04	**lo**
#15	NS3-1406	*0.04*	0.29	0.36	**2.14**	0.35	0.33	**lo**
#15	NS3-1073	*0.03*	0.38	0.32	**0.62**	0.36	0.37	**hi**
#16	NS3-1073	*0.028*	0.37	**2.10**	0.51	0.97	1.29	**hi**
#17	core132	*0.015*	0.69	**4.10**	0.54	0.77	0.60	**lo**
#18	NS3-1073	*0.12*	2.72	3.93	**7.74**	4.29	2.71	**lo**
#19	NS3-1073	*0.02*	0.59	**0.85**	0.81	0.76	0.54	**hi**

*Ex vivo* frequencies and percentages of HCV-specific CD8+ T cells are displayed for all 24 cell lines, for which tetramer-positive cells were detectable after the 10 days culture. 2B4 expression level according to the *ex vivo* MFI are indicated as ‘lo’ or ‘hi’. Responses to peptide stimulation alone are marked in grey. All culture conditions with an at least 2fold increase of tetramer-positive cells as compared to peptide stimulation alone are indicated by black boxes. The highest frequency of tetramer-positive CD8+ T cells detected in each cell line is indicated in bold numbers.

We also analyzed the effect of blocking 2B4 instead of cross-linking on the expansion of HCV-specific CD8+ T cells from chronic hepatitis C patients using a different anti-2B4 antibody. In this setting, we were also able to achieve an increased proliferation of HCV-specific T cells after 10 days of culture in some individuals. Of note, in those cases where the expansion of HCV-specific T cells increased upon 2B4 blockade, no effect or even a negative effect was induced through 2B4 cross-linking (see online supplementary Figure S3). Similarly, when 2B4 cross-linking resulted in an enhanced proliferation of HCV-specific cells, no effect could be seen with blocking 2B4.

### Expression of the 2B4 adaptor protein SAP

**Figure 5:**
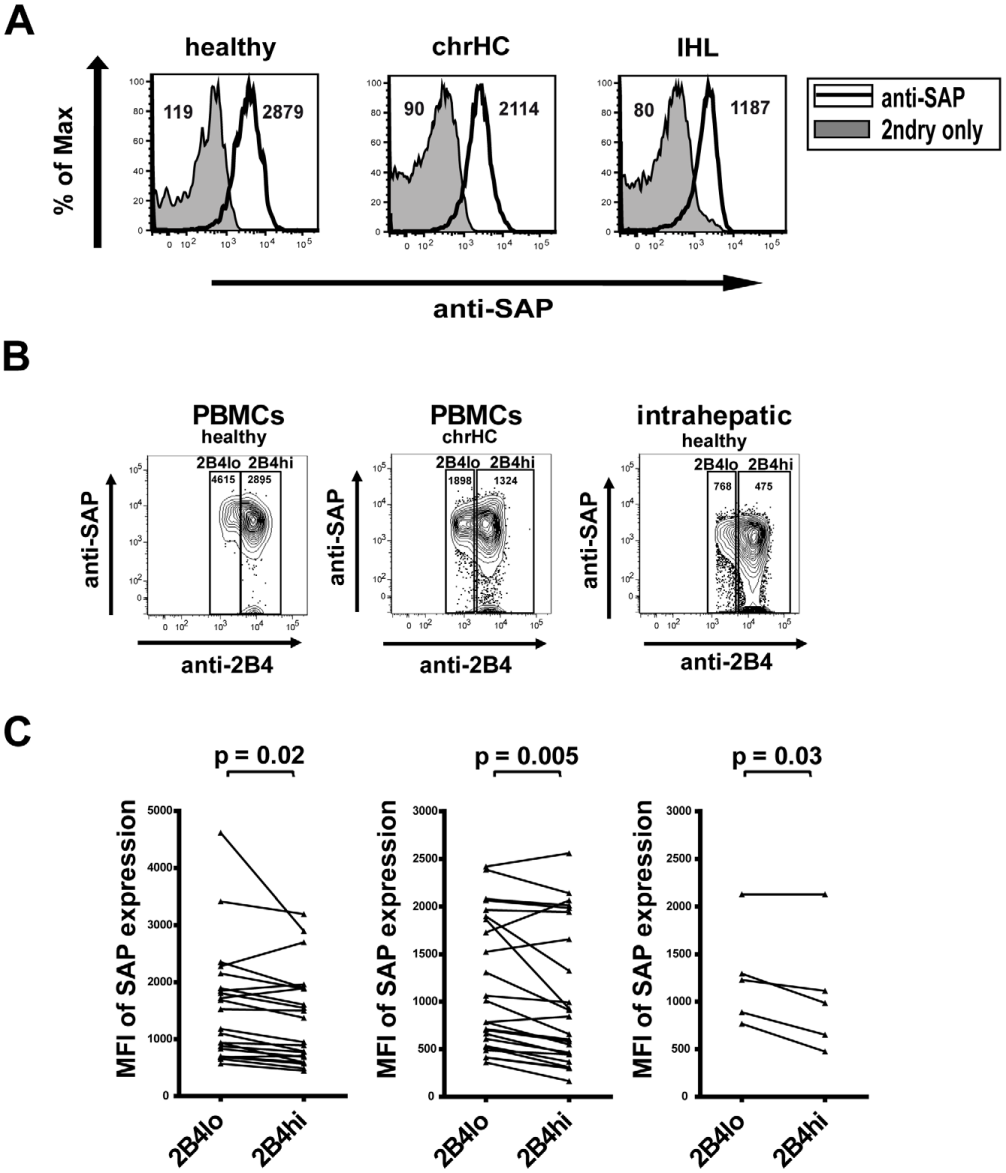
Expression of SAP by CD8+ T cells. Intracellular staining for the 2B4 adaptor molecule SLAM-associated protein (SAP) was performed. (**A**) Representative overlays of histograms for SAP staining (solid line) and respective 2ndry antibody only (tinted graphs) of peripheral CD8+ T cells from healthy individuals (upper left), chronic hepatitis C patients (upper right) and isolated intrahepatic CD8+ T cells (lower left) are shown. MFI values for both histograms are indicated. (**B**) Intracellular SAP content of CD8+ T cells differs with expression intensity of 2B4. Peripheral blood CD8+ T cells from healthy individuals (left plot) and chronic hepatitis C patients (middle plot) and intrahepatic CD8+ T cells (right plot) were stained for 2B4 and intracellular SAP. Cells were gated according to 2B4 expression levels into 2B4lo and 2B4hi cells as shown and content of intracellular SAP was determined by calculating the SAP MFI as indicated. SAP content was found to be lower in cells with high as compared to cells with low 2B4 expression levels. (**C**) Intracellular SAP staining of 2B4+ CD8+ T cells showed a significantly lower SAP expression intensity high 2B4 expression (2B4^hi^) as compared to cells with low 2B4 expression (2B4^lo^) in healthy volunteers (left plot; p = 0.02), patients with chronic hepatitis C (middle plot; p = 0.005) and intrahepatic cells (right plot; p = 0.03).

We next asked whether the variability in the effect of 2B4 stimulation might also be influenced by the signalling pathways elicited. 2B4 ligation can lead to the recruitment of different intracellular adaptor proteins to the cytoplasmic domain of 2B4. As the binding of the adaptor molecule SAP leads to a positive signalling and activation of the cell and as the outcome of 2B4 ligation seems to be dependent on the availability of intracellular SAP molecules as described by Chlewicki et al. [20], we analyzed the expression of SAP in CD8+ T cells in order to elucidate whether the role of 2B4 during CD8+ T cell function and functional exhaustion might be based on differences in SAP expression. For this we used PBMCs from healthy individuals and patients with chronic hepatitis C as well as isolated intrahepatic lymphocytes and stained for intracellular SAP (Figure 5a). No striking differences between SAP expression in peripheral CD8+ T cells from healthy individuals and patients with chronic hepatitis C could be observed. However, SAP contents in T cells isolated form liver tissue tended to be lower as compared to peripheral lymphocytes (Figure 5b). Moreover and importantly, SAP expression differed between 2B4^hi^ and 2B4^lo^ CD8+ T cells as SAP levels were significantly lower in 2B4^hi^ cells in both healthy individuals (MFI 1361±821 vs. 1566±994; p = 0.02), chronic hepatitis C patients (MFI 1023±734 vs. 1170±698; p = 0.005) and intrahepatic T cells (MFI 807±255 vs. 1045±222; p = 0.03; see Figure 5b and 5c). Thus, these findings might explain why HCV-specific CD8+ T cells with high 2B4 expression levels showed a reduction of proliferation upon 2B4 cross-linking, as the reduced intracellular SAP availability results in inhibitory signalling.

### Opposing effects of PD1 blockade and 2B4 cross-linking on the proliferation of HCV-specific CD8+ T cells

**Figure 6:**
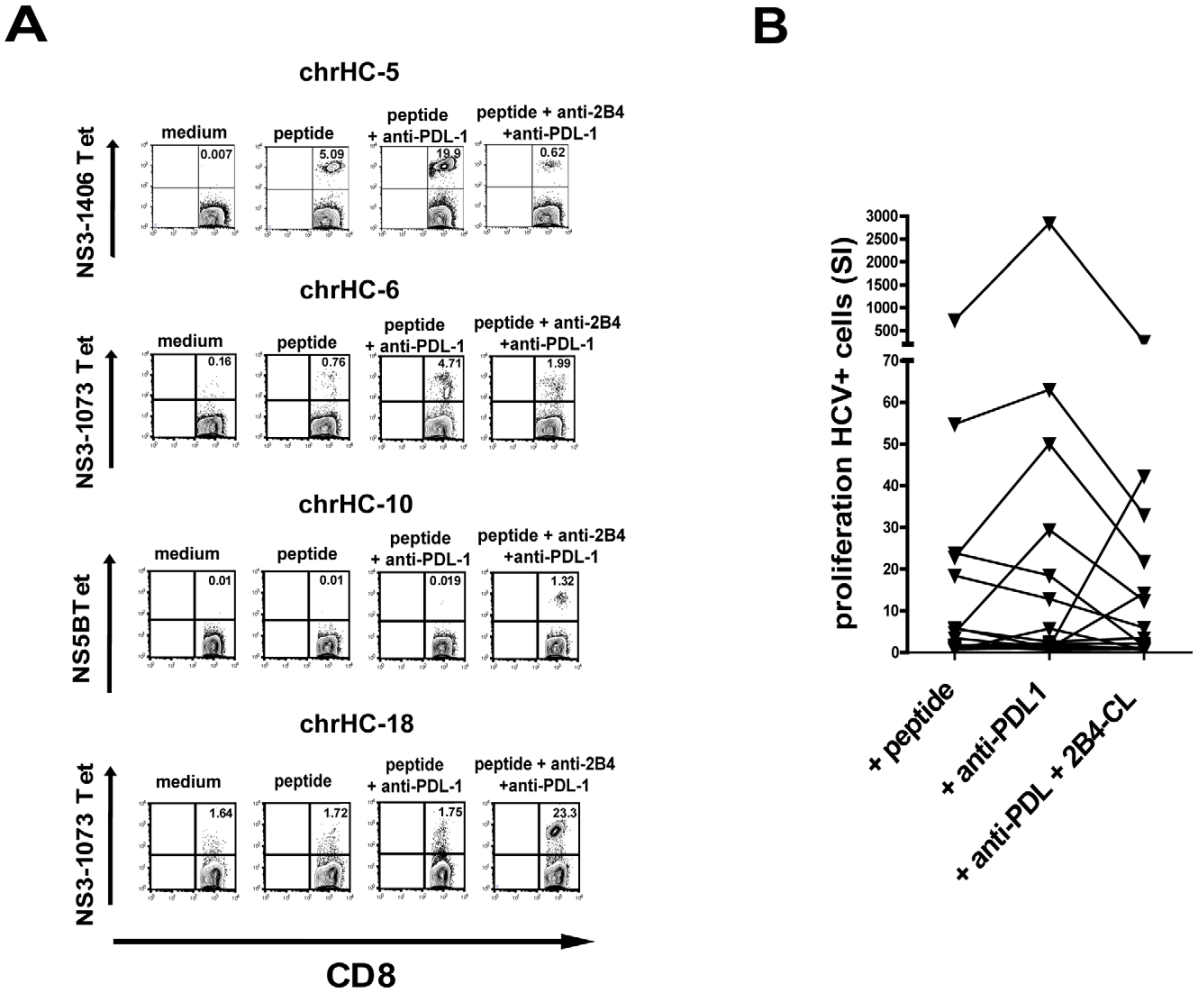
Effect of PD1 blockade and simultaneous cross-linking of 2B4 on the proliferation of HCV-specific CD8+ T cells in patients with chronic hepatitis C. PBMC were stimulated *in vitro* for 10 days with peptide alone or together with the indicated antibodies or antibody combinations. (A) Tetramer staining of three representative cell lines are shown, numbers indicated represent percentages of HCV-tetramer+ cells of CD8+ T cells. (B) Overview of all cell lines analyzed (n = 20), stimulation indices (SI) of percentages of HCV-tetramer+ CD8+ T cells referring to medium controls are given for samples stimulated with peptide alone, additional PD1 blockade or simultaneous PD1 blockade and 2B4 cross-linking. In most cases cross-linking 2B4 in combination with PD1 blockade counter-acted the enhanced proliferation of HCV-specific CD8+ T cells seen upon PD1 blockade alone (6 out of 20). In some cases (3 out of 20) the double combination resulted in enhanced proliferation of HCV-specific CD8+ T cells, in those cases however, blocking PD1 or stimulating 2B4 alone was ineffective.

It has been shown before that blockade of PD1 *in vitro* can enhance HCV-specific CD8+ T cell proliferation and thereby restore functionality of exhausted cells during persistent HCV infection [23], [24]. Similarly, we also observed an increase of HCV-tetramer positive CD8+ T cells by addition of anti-PDL-1 in our cell culture system with 6 out of 19 cell lines (31%) showing a significant increase of tetramer-positive cells as compared to peptide stimulation alone (see table 1). However, as also seen in other reports the susceptibility to PD1 blockade showed a strong inter-individual variability as no positive effect was observed in several individuals analyzed (Figure 6a and 6b). This finding again underlines that PD1 is not alone responsible for the functional exhaustion of virus-specific CD8+ T cells during persistent infections.

Recently, it was shown that multiple costimulatory receptors are involved in the regulation of virus-specific CD8+ T cell function during persistent infection in mice [25]. We therefore investigated the effects of simultaneous PD1 blockade and 2B4 cross-linking on the proliferation of HCV-specific CD8+ T cells. Interestingly, we were able to observe an opposing responsiveness to PD1 blockade and 2B4 stimulation. Those samples positively responding to 2B4 cross-linking did not show an increased proliferation induced by PD1 blockade. Similarly, 2B4 cross-linking alone was ineffective in those cases where proliferation increased upon PD1 blockade (data not shown). Additionally and importantly, 2B4 stimulation was able to counteract enhanced proliferation of HCV-specific CD8+ T cells induced by PD1 blockade. The observed increase of HCV-specific proliferation was abrogated if 2B4 was cross-linked simultaneously (Figure 6a and 6b). Only in a few samples (3 out of 19) the double combination of 2B4 cross-linking and PD1 blockade resulted in enhanced proliferation of HCV-specific CD8+ T cells. In these cases however, neither 2B4 cross-linking nor PD1 blockade alone showed any effect.

## Discussion

In this paper we demonstrate that (i) 2B4 is expressed on virus-specific CD8+ T cells during acute and chronic hepatitis C, (ii) that 2B4 cross-linking can lead to both inhibition and activation of HCV-specific CD8+ T cell function, depending on expression levels of 2B4 and the intracellular adaptor molecule SAP and (iii) that 2B4 stimulation may counteract enhanced proliferation of HCV-specific CD8+ T cells induced by PD1 blockade.

2B4 has first been described on human NK cells [26] and previous studies showed that 2B4 can also be expressed on human CD8+ T cells [27]. Recently, 2B4 expression has been demonstrated also in patients with chronic hepatitis C [22]. We here describe a rather high inter-individual variability of 2B4 expression on CD8+ T cells ranging from less than 10% of cells expressing 2B4 to more than four fifths being 2B4 positive. The majority of 2B4+ CD8+ T cells were differentiated effector memory cells being CD45RA positive and CCR7 negative, although 2B4 expression was not exclusively linked to a specific memory subtype. Nevertheless, further phenotyping showed that the expression of several other costimulatory molecules including PD1 was almost always accompanied by simultaneous 2B4 expression.

During persistent virus infections CD8+ T cells can be functionally impaired and may express high levels of PD1 which control CD8+ T cell functions [9], [23], [28]. Recent data generated in mouse models of chronic viral infections suggested that not only single but multiple coinhibitory molecules are involved in the control of exhausted virus-specific CD8+ T cells [25]. Gene array analysis revealed that, along with others, also 2B4 was significantly upregulated in exhausted virus-specific CD8+ T cells [15]. In line with these findings, we also could demonstrate that HCV-specific CD8+ T cells show an elevated expression of 2B4 in chronic hepatitis C as compared to the respective individual's bulk CD8+ T cells. Further, we could show that expression levels of 2B4 are higher on HCV-specific CD8+ T cells as compared to the respective individual's bulk CD8+ T cells in chronic hepatitis C patients suggesting that 2B4 might be involved in regulating T cell effector functions in chronic infections. In addition, the increased frequency of 2B4+ Flu-specific CD8+ T cells in chronic hepatitis C suggest that cytokines in the context of chronic hepatitis C may result in an unspecific upregulation of 2B4 on Flu-specific CD8+ T cells. Another explanation could be a cross-reactive stimulation of Flu-specific T cells in HCV infected individuals [29]. Moreover, viral escape leading to sub-optimal T cell receptor stimulation may in contrast be associated with a lower 2B4 expression as 2B4 levels were usually low in those subjects showing viral sequence variations in the respective epitopes. Similar findings have previously been shown for other costimulatory molecules [22], [30].

Cross-linking 2B4 using the C1.7 antibody has been shown to stimulate cytotoxicity and cytokine production by NK cells [31]. In contrast, CD8+ T cell function was not altered if 2B4 alone was engaged without simultaneous TCR stimulation [32], [33] which is in line with our own findings (data not shown). Still, a previous report suggested that 2B4 costimulation may enhance *in vitro* expansion of tumour-specific CD8+ T cells [33]. We here show that 2B4 cross-linking may indeed alter effector functions of bulk and antigen-specific CD8+ T cells like proliferation, degranulation and IFNγ production. Further and importantly, 2B4 stimulation was able to enhance peptide-induced proliferation of HCV-specific CD8+ T cells from patients with chronic hepatitis C. Preliminary data from patients with acute hepatitis C confirmed the findings of the stimulatory capacity of 2B4 cross-linking (data not shown). However, this activating effect was only observed in some but not all patients. We noted that responsiveness to 2B4 stimulation was linked with the *ex vivo* 2B4 expression levels, which held true for HCV-specific CD8+ T cells as well as for CMV-, EBV- and Influenza-specific CD8+ T cells. Virus-specific CD8+ T cells with high 2B4 expression levels appeared to be insensitive towards 2B4 stimulation or even showed a decrease in proliferation upon 2B4 cross-linking, while cells with lower 2B4 expression still responded to 2B4 cross-linking. Future studies also need to address in more detail the effects of 2B4 blockade versus 2B4 cross-linking. 2B4 blockade may enhance effector functions of HBV-specific CD8+ T cells in some patients with acute hepatitis B [34]. We identified a positive effect of 2B4 blockade preferentially in those samples with higher 2B4 expression, while 2B4 cross-linking was effective in 2B4-low expressing cells only.

The cause for these opposing functions of 2B4 could be explained with the diverse signal transduction pathways elicited upon 2B4 cross-linking which may lead to a dual function of 2B4. Different adaptor molecules including SAP can be recruited to the intracellular tail of 2B4 [33], [35]. Further studies showed that the expression density of 2B4 influences the outcome of 2B4 signalling. While cell activation seemed to correlate to low 2B4 levels, high 2B4 expression and insufficient availability of SAP molecules lead to an inhibition of cells [20]. These data would explain our findings where 2B4 responsiveness was depended on the level of 2B4 expression. Indeed, we could show that SAP expression was lower in 2B4-high versus 2B4-low expressing CD8+ T cells. The low SAP content in 2B4-high cells may induce inhibitory downstream signalling. In this setting, further stimulation would not result in functional enhancement or might even induce reduction of effector functions. This would also explain the important finding that 2B4 cross-linking counteracted stimulatory effects induced by PD1 blockade. Of note, stimulatory effects by 2B4 cross-linking versus PD1 blockade seemed to be rather exclusive as either one of the mechanisms was only active alone and synergistic effects were rarely observed. Since blocking PD1 is currently explored already in clinical trials our findings may partially question this therapeutic concept as PD1 positive cells are always 2B4 positive and as 2B4 ligands are ubiquitously expressed.

Another 2B4 adaptor molecule is EAT-2, which is supposed to confer inhibitory downstream signalling [36]. Future studies should therefore also consider EAT-2 expression in relation to SAP expression. However, one has to consider that not only the presence of the signal transduction molecules, but also the phosphorylation status of the intracellular domain of 2B4 is of importance [37], potentially explaining the high inter-individual variability observed in our study.

Obviously future studies should address several additional issues. We here provide only first data supporting the hypothesis that 2B4 plays a role in the regulation of CD8+ T cell functions. However, the sample sizes are too low to draw definite conclusions and thus confirmatory studies are needed. Only two co-activating or co-inhibitory receptors potentially regulating CD8+ T cells were investigated and thus additional costimulatory molecules such as CTLA-4, TIM-3 or BLIMP-1 [38], [39] need to be explored in this context. Moreover, we do not provide data on intrahepatic HCV-specific T cell responses. However, almost all IHLs show very high 2B4 expression levels. Thus, we would expect that IHLs should show similar response patterns as peripheral blood 2B4-high HCV-specific CD8+ T cells.

In summary we suggest that 2B4 could be both a marker of CD8+ T cell dysfunction and a potential target for immunointerventions. These findings might be of importance for future development of novel therapies for chronic HCV aiming to achieve immune control.

## Methods

### Patient material

Heparinized peripheral whole blood was collected from acute hepatitis B virus (HBV) and hepatitis C virus (HCV) infected patients (n = 6 and n = 13, respectively) as well as from persistently HCV infected patients (n = 86). Healthy volunteers or samples retrieved from the internal blood donation centre (n = 49) were used as controls. Liver tissue was derived from tumour patients who underwent partial liver resection for evaluation of potential liver metastases or from PSC patients.

### Ethics approval

Written informed consent was obtained from all patients. Ethics approval for this study was obtained by the local ethics committee of Hannover Medical School. All patients were seen in the outpatient clinic of the Department for Gastroenterology, Hepatology and Endocrinology at Hannover Medical School, Germany. Written informed consent was obtained from all patients involved in this study.

### Preparation of PBMCs and intrahepatic lymphocytes

Isolation of peripheral blood mononuclear cells (PBMC) was performed using standard Ficoll Density Centrifugation method. Intrahepatic lymphocytes (IHL) were isolated from liver tissue samples by mechanical disruption through a 70 µm nylon mesh and separated by density centrifugation after washing.

### Monoclonal antibodies and MHC class I complexes

Mouse anti-human fluorochrome-conjugated monoclonal antibodies were purchased as follows: anti-2B4 clone C1.7 (Beckman Coulter, Fullerton, CA, USA), anti-CCR7 (R&D Systems, Minneapolis, MN, USA), anti-PD1 (BioLegend Inc., San Diego, CA, USA), rat anti-SAP and secondary anti-rat IgG (Cell Signaling Technology, Danvers, MA, USA). All other antibodies and mouse IgG isotype controls were obtained from BD Pharmingen (Becton Dickinson, Heidelberg, Germany). For *in vitro* blocking and cross-linking experiments the following monoclonal antibodies were used at a concentration of 5 µg/ml each: purified anti-2B4 (clone C1.7, Beckman Coulter, Fullerton, CA, USA), functional grade purified anti-2B4 (clone eBioPP35), functional grade purified anti-PDL-1 (clone MIH1) and functional grade purified mouse IgG1 isotype control (eBioscience, San Diego, CA, USA). PE-labelled HLA-A*0201 restricted iTag MHC Class I Tetrameric Complexes (tetramers) specific for CMV-pp65 495–504 (NLVPMVATV), EBV-BMLF1 259–267 (GLCTLVAML), influenza A Matrix (influenza-A (IV)) Protein 58–66 (GILGFVFTL), HCV-NS3 1073-1082 (NS3–1073) derived peptide (CINGVCWTV)- HCV-NS3 1406–1415 (KLVALGINAV), HCV-core 35–44 (YLLPRRGPRL), HCV-core 132–140 (ADLMGYIPLV), HCV-NS4B 1789–1796 (SLMAFTAAV), HCV-NS5A 2252–2260 (ILDSFDPLV), HCV-NS5B 2594–2602 (ALYDVVTKL) and HCV-E2 614v621 (RLWHYPCTV) were purchased from Beckman Coulter Inc. (Fullerton, CA, USA). Tetramer staining was considered positive if a distinct population of positive cells could be discriminated. Moreover, at least 0.02% of CD8+ T cells were required to be considered as positive.

### Synthetic MHC class I peptides

Antigenic HLA-A*0201 restricted peptides were purchased from ProImmune Ltd. (Oxford, UK). Peptides were dissolved in sterile endotoxin-free DMSO (Sigma-Aldrich, Munich, Germany) as stock solution. Purity of all peptides was >98%. Final DMSO concentration during T cell culture never exceeded 0.1%. Amino acid sequences of the specific peptides are identical to those of the respective MHC Class I Tetrameric Complexes used.

### Analysis of PBMC by flow cytometry

Expression analysis of 2B4 on lymphocytes was performed directly *ex vivo* after PBMC isolation and detection of antigen-specific CD8+ T cells was performed as described elsewhere [40]. Appropriate unstained and FMO (fluorescence minus one) controls were performed for adjustment of gating. Samples were analysed on a flow cytometer (FACSCalibur or FACSCantoII, Becton Dickinson, Heidelberg, Germany) within 30 minutes. Analysis of FACS data was performed using FlowJo Software (TreeStar Inc., San Diego, CA, USA).

### *In vitro* culture of T cells

Frozen PBMCs were resuspended in RPMI-1640 (Invitrogen, Karlsruhe, Germany) supplied with 10% human AB-Serum (Cambrex, East Rutherford, NJ, USA), non-essential amino acids and sodium private (Invitrogen, Karlsruhe, Germany), 2 µM HEPES (Invitrogen, Karlsruhe, Germany) and Penicillin/Streptomycin (100U/ml Penicillin and 100 µg/ml Streptomycin; PAA, Pasching, Austria). 3×10^5^ PBMCs per well and condition were stimulated in 96-well U-bottom plates (Sarstedt GmbH, Nümbrecht, Germany) at 37°C and 5% CO_2_. Experiments were set up in multiple replicates if possible. Medium alone or peptide alone with IgG1 isotype control antibodies served as a negative control. Respective antigenic peptides were added at optimal concentrations as mentioned above.

### Enumeration of antigen-specific CD8+ T cells after *in vitro* culture

The frequency of antigen-specific CD8+ T cells tetramer staining was analyzed after *in vitro* expansion of cells after seven days in healthy individuals and after ten days for chronic HCV patients. Human recombinant IL-2 (Invitrogen, Karlsruhe, Germany) was added at day 3 or 5 in concentrations of 5U/ml, respectively. For healthy individuals changes in proliferation of virus-specific CD8+ T cells was analyzed by calculating the stimulation index (SI) of samples with peptide+2B4 cross-linking in relation to peptide stimulation alone. Grouping of cell samples into 2B4-low and 2B4-high was done according to the ex vivo expression levels (MFI) of 2B4 on the respective tetramer-positive CD8+ T cell. Cut-offs were set according to the average 2B4 MFI calculated for all tetramer-positive CD8+ T cells analyzed, respectively.

### CFSE proliferation assay

Proliferation of bulk and antigen-specific CD8+ T cells was analyzed by CFSE staining exactly as described previously [40]. Cells were stained with 4 µM CFSE prior to culture. After 7 days of *in vitro* culture the percentage of dividing CFSE-low cells was analyzed by flow cytometry.

### Analysis of CD8+ T cell effector functions

Degranulation (CD107a/b expression) as a surrogate marker for cytotoxicity was analyzed after *in vitro* stimulation of PBMC by flow cytometry as previously described [40]. In addition, 2B4+/CD8+ and 2B4-/CD8+ T cells were sorted by flow cytometry (BD FACSAria, Becton Dickinson, Heidelberg, Germany), incubated with anti-CD3/28 beads and stained for CD107a/b. IFNγ production of CD8+ T cells was investigated by intracellular cytokine staining after 6h *in vitro* stimulation with peptides or anti-CD3/28 beads and analyzed by flow cytometry [40].

### Annexin-V staining

Determination of the viability of CD8+ T cells was performed after 3 days *in vitro* culture by staining for Annexin-V using the Annexin-V Staining Kit (Becton Dickinson, Heidelberg, Germany) according to manufacturer's protocol. PBMC from healthy individuals were stimulated by anti-CD3/28 and additional 2B4 cross-linking.

### Intracellular staining for SAP

Intracellular staining of the 2B4 signalling adaptor molecule SLAM-associated protein (SAP) was performed in peripheral or intrahepatic lymphocytes using the BD CytoPerm/Wash Buffer Kit (BD Pharmingen). After surface staining cells were fixed and following permeabilization stained using anti-SAP antibody, detection was performed using a secondary antibody. Cells were then analyzed on a flow cytometer.

### Statistical analyses

For descriptive means statistics are expressed as mean values ± standard deviations. Statistical analysis of stimulation experiments were performed using considered two-tailed unpaired Student's T tests. Increase of effector functions were considered significant if the calculated SI referring to the medium or peptide only sample were ≤2.0. Man-Whitney U-Tests were used for analyzing differences in 2B4 expressions. For calculating differences in responsiveness of chronic hepatitis C patients to peptide stimulation or additional 2B4 cross-linking or PD1 blockade a simple Chi-Square Test was used. *P* values of <0.05 were considered as significant.

### Accession numbers

Accession Numbers and IDs of human proteins referred to in this manuscript were received from UniProt (http://www.ebi.ac.uk/uniprot/)

**2B4** (alternative names: CD244) Accession Number Q9BZW8
**CD48** (alternative names: B-lymphocyte activation marker BLAST-1) Accession Number P09326
**SAP** (SLAM-associated protein, alternative name: SH2 domain-containing protein 1A, SH2D1A) Accession Number: O60880
**EAT-2** (EWS/FLI1-activated transcript 2, alternative name: SH2 domain-containing protein 1B, SH2D1B) Accession Number: O14796
**PD1** (Programmed cell death protein 1, alternative names: CD279, PDCD1) Accession Number: Q15116
**PDL-1** (Programmed cell death 1 ligand 1, alternative names: CD274, PDCD1L1) Accession Number: Q9NZQ7

## Supporting Information

Figure S1 Degranulation of sorted 2B4+/CD8+ and 2B4-/CD8+ T cells. PBMCs were sorted into 2B4+ (white bars) and 2B4- CD8+ (grey bars) T cells and stimulated in vitro for analyzing their degranulation. Only 2B4+ CD8+ T cells showed an increased expression of CD107a/b. Stimulation indices (SI) referring to medium control are given; n = 7.
(TIF)

Figure S2 Annexin-V expression after 2B4 cross-linking. Cells were stained for Annexin-V content after 3 days stimulation of PBMCs with or without anti-CD3/28 and with or without 2B4 cross-linking in vitro. No difference in Annexin-V expression upon additional 2B4 cross-linking could be seen. Percentages of Annexin-V positive CD8+ T cells are given; n = 5.
(TIF)

Figure S3 Effects of 2B4 cross-linking versus 2B4 blockade on the expansion of HCV-specific CD8+ T cells. Expansion of HCV-specific CD8+ T cells from chronic hepatitis C patients upon peptide stimulation and additional 2B4 cross-linking or 2B4 blocking was analyzed by tetramer-staining after 10 days. Responsiveness towards 2B4 cross-linking or 2B4 blockade varied with 2B4 expression levels on tetramer-positive cells *ex vivo*. FACS plots of three representative cell lines are shown, frequencies of tetramer-positive cells are indicated. Cells were gated on CD14/CD19/CD56-negative and CD8+ T cells.
(TIF)

Table S1 2B4 expression levels and autologous viral sequences. Expression levels of 2B4 on CD8+ T cells specific for the HCV NS3-1073 and NS3-1406 epitopes were analyzed, frequency and mean fluorescence intensities (MFI) of 2B4 are indicated. Autologous viral sequences of these two epitopes were analyzed by sequencing in order to identify viral escape mutations. Deviations from the wild type sequences used for peptides and tetramers are indicated in bold and underlined, the respective HCV genotype (HCV GT) is given.
(TIF)

## References

[1] Whitmire JK, Ahmed R (2000). Costimulation in antiviral immunity: differential requirements
for CD4(+) and CD8(+) T cell responses.. Curr Opin Immunol.

[2] Seeff LB (2002). Natural history of chronic hepatitis C.. Hepatology.

[3] Rehermann B, Nascimbeni M (2005). Immunology of hepatitis B virus and hepatitis C virus
infection.. Nat Rev Immunol.

[4] Bowen DG, Walker CM (2005). Mutational escape from CD8+ T cell immunity: HCV evolution,
from chimpanzees to man.. J Exp Med.

[5] Wedemeyer H, He XS, Nascimbeni M, Davis AR, Greenberg HB (2002). Impaired effector function of hepatitis C virus-specific
CD8+ T cells in chronic hepatitis C virus infection.. J Immunol.

[6] Gruener NH, Lechner F, Jung MC, Diepolder H, Gerlach T (2001). Sustained dysfunction of antiviral CD8+ T lymphocytes after
infection with hepatitis C virus.. J Virol.

[7] Grakoui A, Shoukry NH, Woollard DJ, Han JH, Hanson HL (2003). HCV persistence and immune evasion in the absence of memory T
cell help.. Science.

[8] Thimme R, Oldach D, Chang KM, Steiger C, Ray SC (2001). Determinants of viral clearance and persistence during acute
hepatitis C virus infection.. J Exp Med.

[9] Barber DL, Wherry EJ, Masopust D, Zhu B, Allison JP (2006). Restoring function in exhausted CD8 T cells during chronic viral
infection.. Nature.

[10] Wherry EJ, Blattman JN, Murali-Krishna K, van der Most R, Ahmed R (2003). Viral persistence alters CD8 T-cell immunodominance and tissue
distribution and results in distinct stages of functional
impairment.. J Virol.

[11] Kaufmann DE, Walker BD (2009). PD-1 and CTLA-4 inhibitory cosignaling pathways in HIV infection
and the potential for therapeutic intervention.. J Immunol.

[12] Rehermann B (2009). Hepatitis C virus versus innate and adaptive immune responses: a
tale of coevolution and coexistence.. J Clin Invest.

[13] Nakamoto N, Kaplan DE, Coleclough J, Li Y, Valiga ME (2008). Functional restoration of HCV-specific CD8 T cells by PD-1
blockade is defined by PD-1 expression and
compartmentalization.. Gastroenterology.

[14] Kasprowicz V, Schulze Zur Wiesch J, Kuntzen T, Nolan BE, Longworth S (2008). High level of PD-1 expression on hepatitis C virus (HCV)-specific
CD8+ and CD4+ T cells during acute HCV infection, irrespective of
clinical outcome.. J Virol.

[15] Wherry EJ, Ha SJ, Kaech SM, Haining WN, Sarkar S (2007). Molecular signature of CD8+ T cell exhaustion during chronic
viral infection.. Immunity.

[16] McNerney ME, Guzior D, Kumar V (2005). 2B4 (CD244)-CD48 interactions provide a novel MHC class
I-independent system for NK-cell self-tolerance in mice.. Blood.

[17] Brown MH, Boles K, van der Merwe PA, Kumar V, Mathew PA (1998). 2B4, the natural killer and T cell immunoglobulin superfamily
surface protein, is a ligand for CD48.. J Exp Med.

[18] Tangye SG, Cherwinski H, Lanier LL, Phillips JH (2000). 2B4-mediated activation of human natural killer
cells.. Mol Immunol.

[19] Nakajima H, Cella M, Langen H, Friedlein A, Colonna M (1999). Activating interactions in human NK cell recognition: the role of
2B4-CD48.. Eur J Immunol.

[20] Chlewicki LK, Velikovsky CA, Balakrishnan V, Mariuzza RA, Kumar V (2008). Molecular basis of the dual functions of 2B4
(CD244).. J Immunol.

[21] Ma CS, Nichols KE, Tangye SG (2007). Regulation of cellular and humoral immune responses by the SLAM
and SAP families of molecules.. Annu Rev Immunol.

[22] Bengsch B, Seigel B, Ruhl M, Timm J, Kuntz M (2010). Coexpression of PD-1, 2B4, CD160 and KLRG1 on exhausted
HCV-specific CD8+ T cells is linked to antigen recognition and T cell
differentiation.. PLoS Pathog.

[23] Urbani S, Amadei B, Tola D, Pedrazzi G, Sacchelli L (2008). Restoration of HCV-specific T cell functions by PD-1/PD-L1
blockade in HCV infection: effect of viremia levels and antiviral
treatment.. J Hepatol.

[24] Penna A, Pilli M, Zerbini A, Orlandini A, Mezzadri S (2007). Dysfunction and functional restoration of HCV-specific CD8
responses in chronic hepatitis C virus infection.. Hepatology.

[25] Blackburn SD, Shin H, Haining WN, Zou T, Workman CJ (2009). Coregulation of CD8+ T cell exhaustion by multiple
inhibitory receptors during chronic viral infection.. Nat Immunol.

[26] Valiante NM, Trinchieri G (1993). Identification of a novel signal transduction surface molecule on
human cytotoxic lymphocytes.. J Exp Med.

[27] Boles KS, Stepp SE, Bennett M, Kumar V, Mathew PA (2001). 2B4 (CD244) and CS1: novel members of the CD2 subset of the
immunoglobulin superfamily molecules expressed on natural killer cells and
other leukocytes.. Immunol Rev.

[28] Nakamoto N, Cho H, Shaked A, Olthoff K, Valiga ME (2009). Synergistic reversal of intrahepatic HCV-specific CD8 T cell
exhaustion by combined PD-1/CTLA-4 blockade.. PLoS Pathog.

[29] Wedemeyer H, Mizukoshi E, Davis AR, Bennink JR, Rehermann B (2001). Cross-reactivity between hepatitis C virus and Influenza A virus
determinant-specific cytotoxic T cells.. J Virol.

[30] Kasprowicz V, Kang YH, Lucas M, Schulze zur Wiesch J, Kuntzen T (2010). Hepatitis C virus (HCV) sequence variation induces an
HCV-specific T-cell phenotype analogous to spontaneous
resolution.. J Virol.

[31] Chuang SS, Kim MH, Johnson LA, Albertsson P, Kitson RP (2000). 2B4 stimulation of YT cells induces natural killer cell cytolytic
function and invasiveness.. Immunology.

[32] Kambayashi T, Assarsson E, Chambers BJ, Ljunggren HG (2001). Cutting edge: Regulation of CD8(+) T cell proliferation by
2B4/CD48 interactions.. J Immunol.

[33] Altvater B, Landmeier S, Pscherer S, Temme J, Juergens H (2009). 2B4 (CD244) signaling via chimeric receptors costimulates
tumor-antigen specific proliferation and in vitro expansion of human T
cells.. Cancer Immunol Immunother.

[34] Raziorrouh B, Schraut W, Gerlach T, Nowack D, Grüner NH (2010). The immunoregulatory role of CD244 in chronic hepatitis B
infection and its inhibitory potential on virus-specific CD8+ T-cell
function.. Hepatology.

[35] Dong Z, Cruz-Munoz ME, Zhong MC, Chen R, Latour S (2009). Essential function for SAP family adaptors in the surveillance of
hematopoietic cells by natural killer cells.. Nat Immunol.

[36] Veillette A (2006). Immune regulation by SLAM family receptors and SAP-related
adaptors.. Nat Rev Immunol.

[37] Chen R, Relouzat F, Roncagalli R, Aoukaty A, Tan R (2004). Molecular dissection of 2B4 signaling: implications for signal
transduction by SLAM-related receptors.. Mol Cell Biol.

[38] Shin H, Blackburn SD, Intlekofer AM, Kao C, Angelosanto JM (2009). A role for the transcriptional repressor Blimp-1 in CD8(+) T
cell exhaustion during chronic viral infection.. Immunity.

[39] Golden-Mason L, Palmer BE, Kassam N, Townshend-Bulson L, Livingston S (2009). Negative immune regulator Tim-3 is overexpressed on T cells in
hepatitis C virus infection and its blockade rescues dysfunctional CD4+
and CD8+ T cells.. J Virol.

[40] Suneetha PV, Schlaphoff V, Wang C, Stegmann KA, Fytili P (2009). Effect of peptide pools on effector functions of antigen-specific
CD8+ T cells.. J Immunol Methods.

