# Supplementary figures and images for: Dual Function of the NK Cell Receptor 2B4 (CD244) in the Regulation of HCV-Specific CD8+ T Cells
Source: PLoS Pathog. 2011 May 19;7(5):e1002045. doi: 10.1371/journal.ppat.1002045 (PMC3098233; doi:10.1371/journal.ppat.1002045)

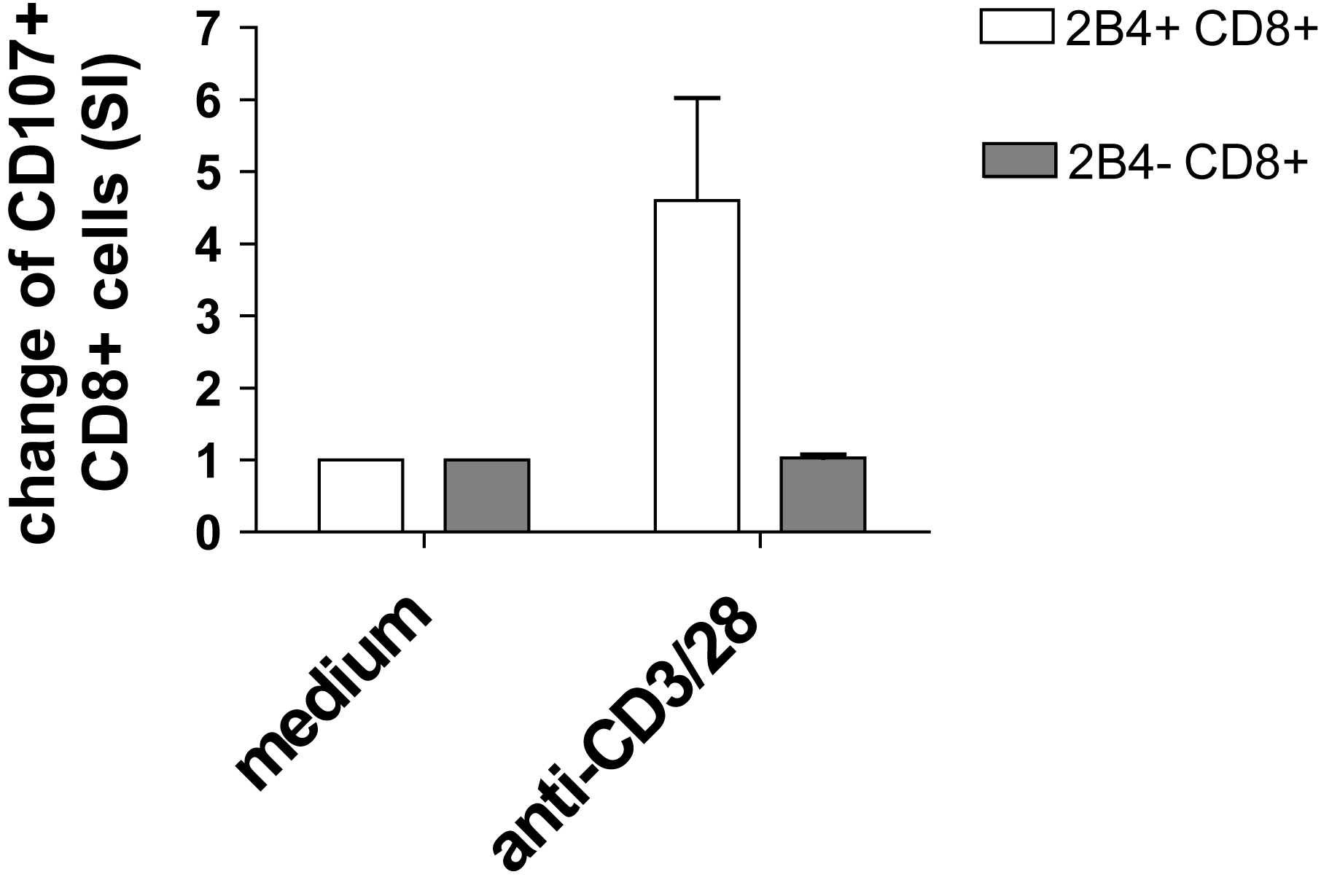

Supplement: Figure S1 — Degranulation of sorted 2B4+/CD8+ and 2B4-/CD8+ T cells. PBMCs were sorted into 2B4+ (white bars) and 2B4- CD8+ (grey bars) T cells and stimulated in vitro for analyzing their degranulation. Only 2B4+ CD8+ T cells showed an increased expression of CD107a/b. Stimulation indices (SI) referring to medium control are given; n = 7. (TIF) [file ppat.1002045.s001.tif]

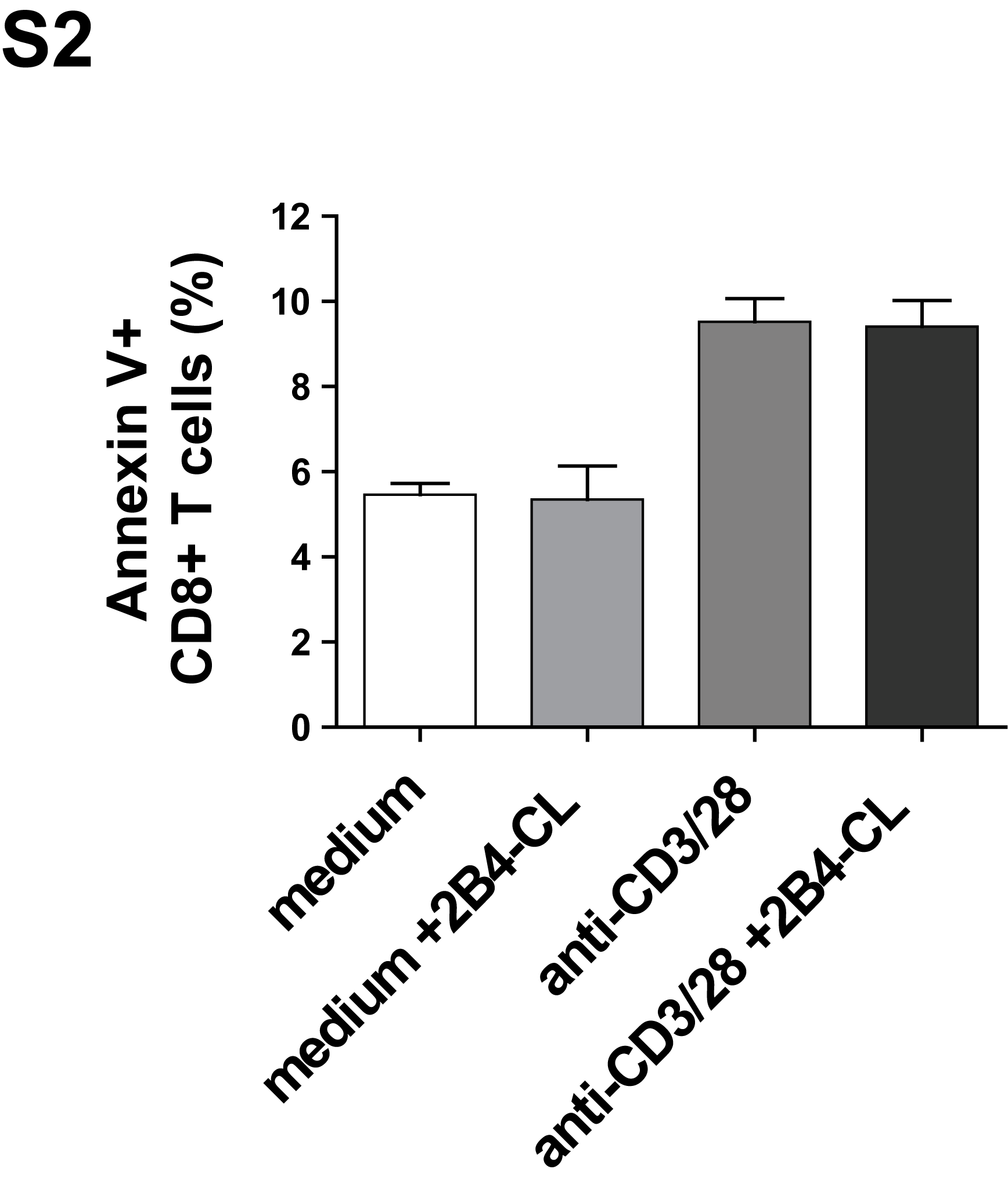

Supplement: Figure S2 — Annexin-V expression after 2B4 cross-linking. Cells were stained for Annexin-V content after 3 days stimulation of PBMCs with or without anti-CD3/28 and with or without 2B4 cross-linking in vitro. No difference in Annexin-V expression upon additional 2B4 cross-linking could be seen. Percentages of Annexin-V positive CD8+ T cells are given; n = 5. (TIF) [file ppat.1002045.s002.tif]

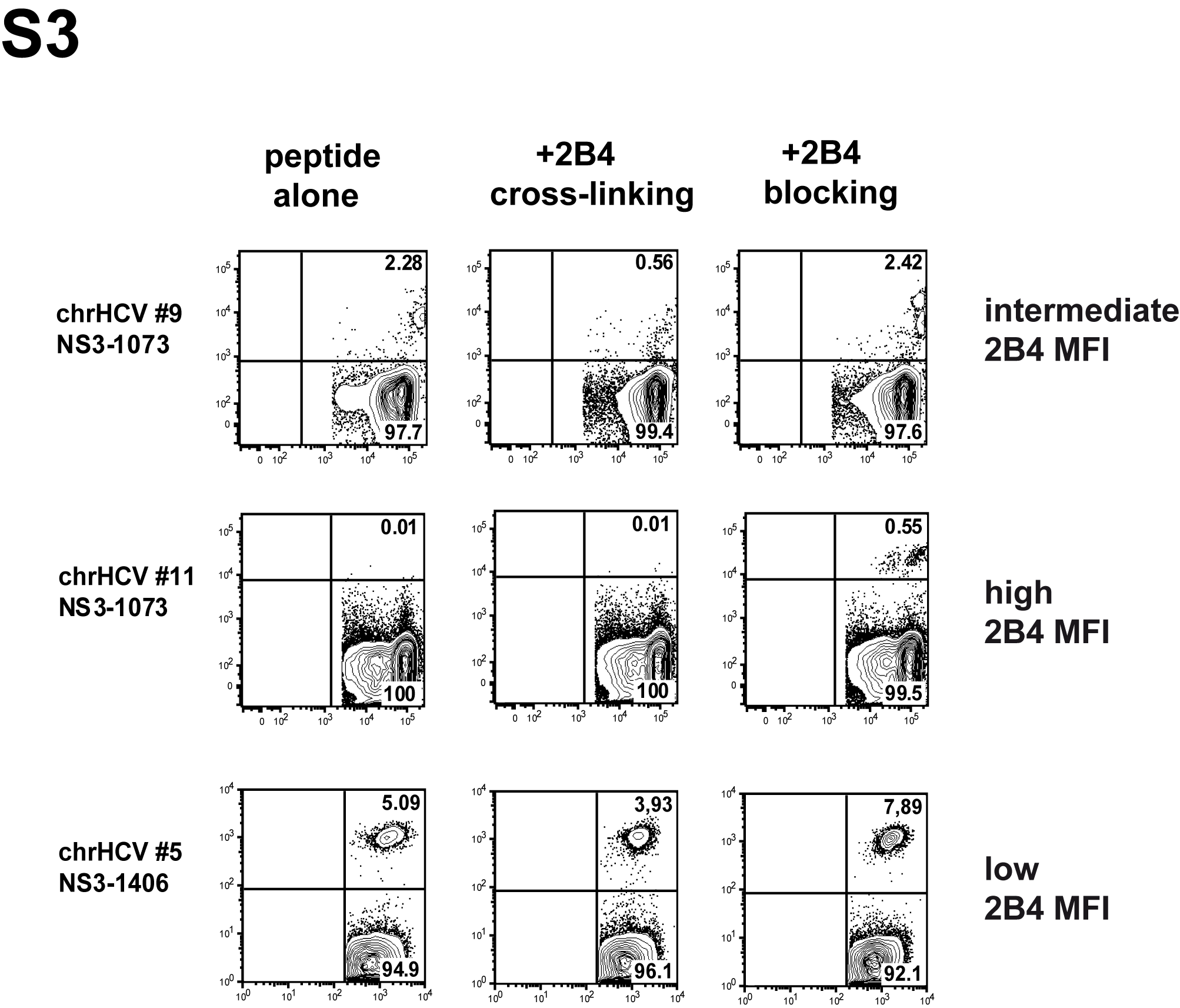

Supplement: Figure S3 — Effects of 2B4 cross-linking versus 2B4 blockade on the expansion of HCV-specific CD8+ T cells. Expansion of HCV-specific CD8+ T cells from chronic hepatitis C patients upon peptide stimulation and additional 2B4 cross-linking or 2B4 blocking was analyzed by tetramer-staining after 10 days. Responsiveness towards 2B4 cross-linking or 2B4 blockade varied with 2B4 expression levels on tetramer-positive cells ex vivo. FACS plots of three representative cell lines are shown, frequencies of tetramer-positive cells are indicated. Cells were gated on CD14/CD19/CD56-negative and CD8+ T cells. (TIF) [file ppat.1002045.s003.tif]

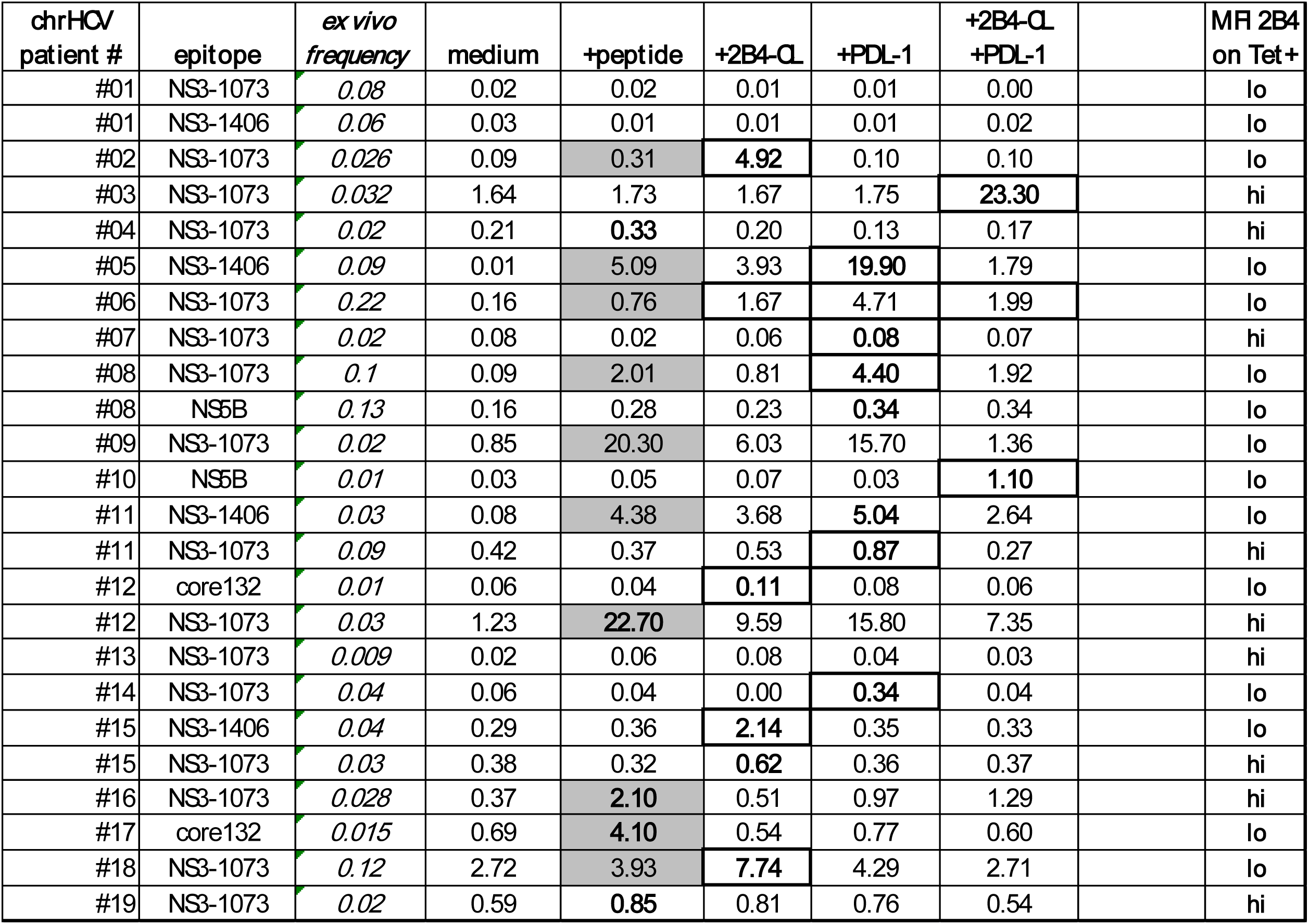

Supplement: Table S1 — 2B4 expression levels and autologous viral sequences. Expression levels of 2B4 on CD8+ T cells specific for the HCV NS3-1073 and NS3-1406 epitopes were analyzed, frequency and mean fluorescence intensities (MFI) of 2B4 are indicated. Autologous viral sequences of these two epitopes were analyzed by sequencing in order to identify viral escape mutations. Deviations from the wild type sequences used for peptides and tetramers are indicated in bold and underlined, the respective HCV genotype (HCV GT) is given. (TIF) [file ppat.1002045.s004.tif]
